# Sudomotor Function as a Tool for Cardiorespiratory Fitness Level Evaluation: Comparison with Maximal Exercise Capacity

**DOI:** 10.3390/ijerph110605839

**Published:** 2014-05-30

**Authors:** Anu Raisanen, Jyrki Eklund, Jean-Henri Calvet, Jaakko Tuomilehto

**Affiliations:** 1 Aino Active (currently Aino Health Management), Pitäjänmäentie 14 4. krs, 00380 Helsinki, Finland; E-Mails: amr@iki.fi (A.R.); jyrki.eklund@ainohealth.com (J.E.); 2 Impeto-Medical, Paris, France, 17 Rue Campagne Première, 75014 Paris, France. E-Mail: jean-henri.calvet@impeto-medical.com; 3 Center for Vascular Prevention, Department for Clinical Neurosciences and Preventive Medicine, Danube-University Krems, Dr.-Karl-Dorrek-Str. 30, 3500 Krems, Austria; 4 Diabetes Prevention Unit, National Institute for Health and Welfare, P.O. Box 30, 00271 Helsinki, Finland; 5 Instituto de Investigacion Sanitaria del Hospital Universario LaPaz (IdiPAZ), 261 28046 Madrid, Spain; 6 Diabetes Research Group, King Abdulaziz University, Abdullah Sulayman, Jeddah 22254, Saudi Arabia

**Keywords:** sweat dysfunction, small fiber neuropathy, lifestyle intervention, cardiometabolic risk, VO_2_max, physical fitness, body mass

## Abstract

Physical inactivity is a modifiable risk factor for cardiovascular (CV) and metabolic disorders. VO_2_max is the best method to assess cardio-respiratory fitness level but it is poorly adopted in clinical practice. Sudomotor dysfunction may develop early in metabolic diseases. This study aimed at comparing established CV risk evaluation techniques with SUDOSCAN; a quick and non-invasive method to assess sudomotor function. A questionnaire was filled-in; physical examination and VO_2_max estimation using a maximal test on a bicycle ergometer were performed on active Finish workers. Hand and foot electrochemical skin conductance (ESC) were measured to assess sudomotor function. Subjects with the lowest fitness level were involved in a 12 month training program with recording of their weekly physical activity and a final fitness level evaluation. Significant differences in BMI; waist and body fat were seen according to SUDOSCAN risk score classification. Correlation between the risk score and estimated VO_2_max was r = −0.57, *p* < 0.0001 for women and −0.48, *p* < 0.0001 for men. A significant increase in estimated VO_2_max, in hand and foot ESC and in risk score was observed after lifestyle intervention and was more important in subjects with the highest weekly activity. SUDOSCAN could be used to assess cardio-metabolic disease risk status in a working population and to follow individual lifestyle interventions.

## 1. Introduction

Obesity and sedentary lifestyle are known risk factors for cardiovascular (CV) and metabolic diseases. Lifestyle intervention trials have demonstrated that prevention of CV disease and type 2 diabetes is possible among high-risk individuals [1,2,3]. The impact of these lifestyle interventions, which are aimed at improving CV and metabolic health, can be assessed by measuring improvements in weight and waist circumference. In addition to the amount of physical activity the level of physical fitness is also important for CV and metabolic health [4]. VO_2_max is a standard and direct measure of fitness and CV health, but is a relatively complex test to perform making it unsuitable for the follow-up of large scale intervention programs [5].

Eccrine glands that are responsible for the sweat response are innervated by a rich supply of blood vessels and sympathetic C nerve fibers. Several studies using quantitative sudomotor axon reflex test (QSART) or sympathetic skin response have shown sudomotor dysfunction in diabetes and prediabetes [6,7]. Skin biopsies confirmed nerve injuries in such conditions and their recovery following lifestyle interventions [8]. However the tests used to assess sudomotor function or skin biopsies are time consuming or invasive [9].

SUDOSCAN a simple and quick test was recently developed to allow measurement of sweat gland function and it has been evaluated in screening for diabetes and pre-diabetes [10,11]. The aim of this study was to compare the health status analysis data for CV risk evaluation (weight, waist circumference, body fat and estimated VO_2_max) with SUDOSCAN measurement in the general working population and to assess the effect of a one year health promotion program including recording of weekly activity proposed to subjects with low fitness level at baseline on all these parameters.

## 2. Experimental Section

The study was conducted from November 2009 to March 2011. Participants were permanent employees of the town of Tornio, Finland. All 1,027 employees were approached to participate in this study. The 650 subjects that gave their informed consent were invited to fill in a questionnaire on medical history and use of medication. After general physical examination including measurements of BMI, waist circumference, body fat percentage and blood pressure a physical test was performed for subjects without exclusion criteria for this exercise. Main exclusion criteria for this exercise test were use of β-blockers, use of more than one antihypertensive drug, systolic blood pressure > 170 mmHg or diastolic blood pressure > 110 mmHg. The maximal test performed on a bicycle ergometer gave prediction of VO_2_max [12]. No heart rate or time limit was imposed and a maximal effort was encouraged. Standardized equations were used to calculate the metabolic equivalents (METs) on the basis of cycle ergometer watts. Exercise capacity was expressed as the maximal MET value attained during the exercise test. Based on the results of physical examination, body fat, and VO_2_max participants were classified into three groups, no risk, moderate or high CV risk. Those classified at the highest risk (any one or more of the following: BMI > 35 kg/m², very low estimated VO_2_max when compared to average range for own age group, diabetes or heart disease) were invited to a supervised health promotion program in small groups adapted for each individual, twice a week for 60 min. Their weekly physical activity was evaluated by the assessment of time spent on moderate or high physical activity during each week between month 6 and 12 after baseline. They had a second fitness level evaluation at the end of the intervention program, *i.e.*, at 12 months.

**Figure 1:**
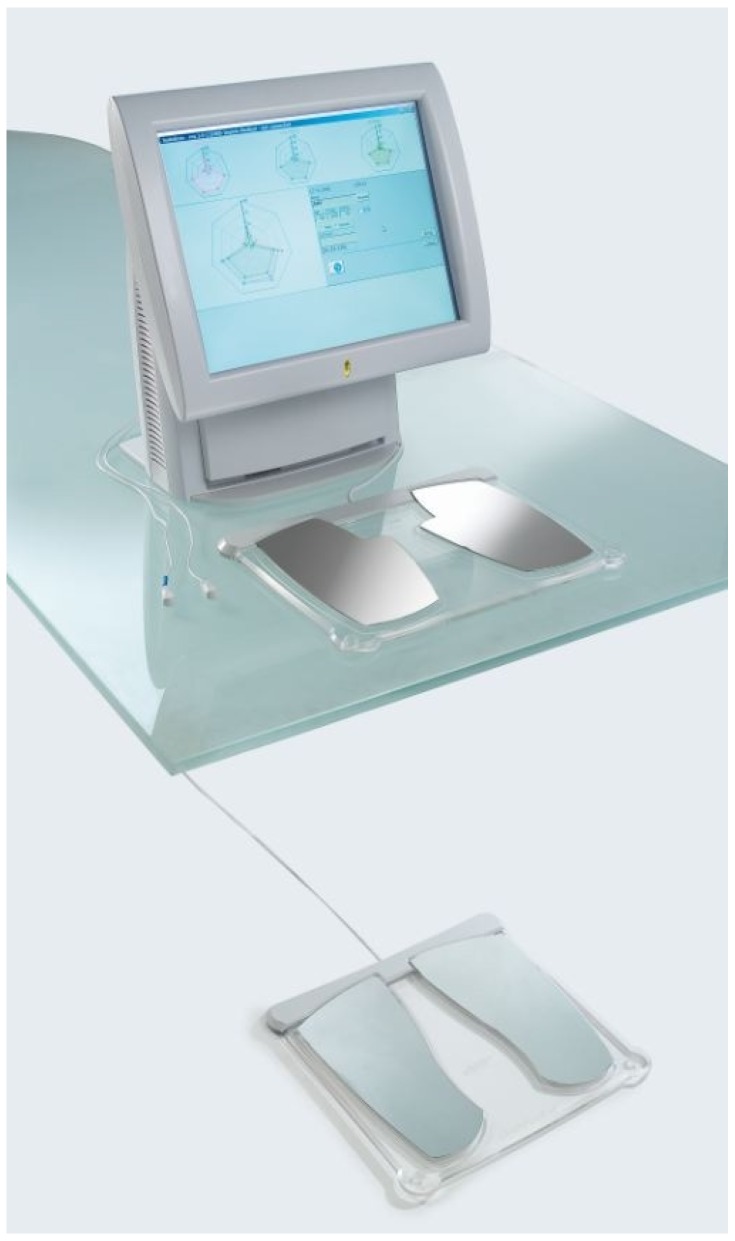
General Presentation of the SUDOSCAN Device with Hand and Foot Electrodes.

Sudomotor function assessment: SUDOSCAN (Impeto Medical, Paris, France) is a new patented device designed to perform a precise evaluation of sweat gland function based on an electrochemical reaction between sweat chlorides and electrodes in contact with hands and feet using reverse iontophoresis and chronoamperometry [10,11]. The apparatus consists of two sets of stainless-steel electrodes in contact with the palms of the hands and soles of the feet where sweat gland density is the most important and connected to a computer for recording and data management purposes (Figure 1).

To conduct the test, the individual is required to stand still for 2 min. During the test four combinations of 15 different low direct current (DC) incremental voltages ≤ 4 V are applied. At low DC voltage, the stratum corneum of the skin constitutes an electrical barrier which prevents any other way of liquid extraction, as shown in the Chizmadzhev model [13].

Neither special subject preparation nor medical personnel with special training is required. The chlorides of the sweat attracted by the electrodes when a DC is applied create a current proportional to their concentration through an electrochemical reaction with the nickel of the electrodes at low DC stimuli. A time/ampere curve is recorded for each derivation. The data, namely electrochemical skin conductance (ESC) in hands and feet, ratio between current generated and the constant DC stimulus, are displayed instantaneously on a PC monitor in the form of a geometric figure that allows fast interpretation. To make the interpretation of the results convenient a risk score based on sweat dysfunction and age is also provided with the following threshold values: ≤ 20 for no risk, [20–30] for moderate risk and ≥ 30 for elevated risk. A corresponding color classification is given to allow an intuitive interpretation by the person undertaking the measurement (green = normal sweat function, no risk; yellow = moderate sweat dysfunction, moderate risk; orange-red: high sweat dysfunction, elevated risk).

Reproducibility of this sweat function measurement had been successfully validated in previous studies [10] and was therefore relied on in this study to confirm the robustness of the evaluation method: the first measurement was performed before bicycle exercise used to assess estimated VO_2_max and the second one performed just after the end of the exercise. The data analysis was performed in a blinded fashion by an independent person.

Laboratory assessment: HbA_1C_ was measured using Siemens DCA Vantage Analyzer (Siemens AG, Erlangen, Germany).

Statistical analyses: Results for quantitative variables are shown as mean ± sd. Due to differences in estimated VO_2_max analyses were performed according to gender. Group means were globally compared using Kruskall-Wallis test. Comparisons between groups were done using Nemenyi-Damico-Wolfe-Dunn test [14]. As a rule, a *p*-value < 0.05 was regarded as statistically significant. Reproducibility was evaluated using Bland-Altman plots with bias and confidence interval and the mean percent difference was calculated [15]. Evaluation of the intervention program was performed only in women due to small number of men involved in this study.

## 3. Results and Discussion

**Figure 2:**
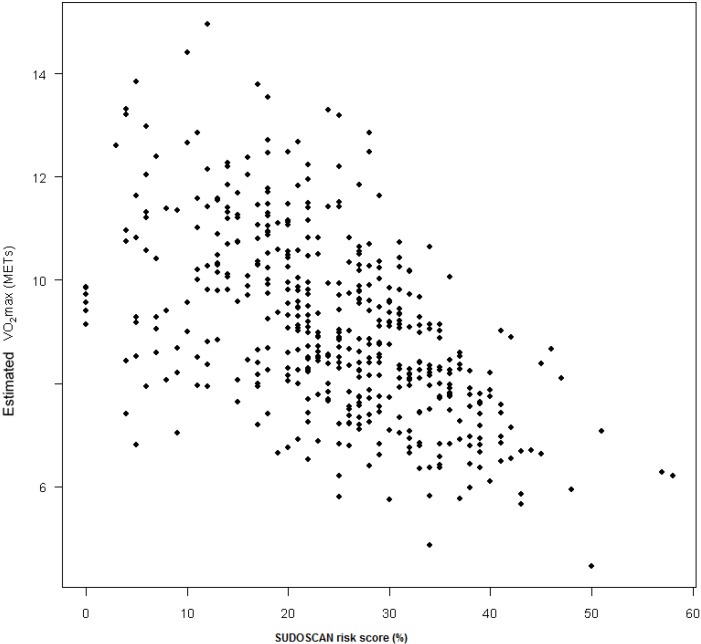
Correlation between SUDOSCAN risk score based on sudomotor dysfunction and estimated VO_2_max in women (*n* = 537).

This study performed in active workers showed that correlation between SUDOSCAN risk scale and estimated VO_2_max was *r* = ‒0.57, *p* < 0.0001 for women (Figure 2) and *r* = ‒0.48, *p* < 0.0001 for men. Baseline characteristics of the entire population and according to gender and SUDOSCAN risk score are displayed in Table 1. Based on Bland-Altman plots for reproducibility between a first measurement performed before the bicycle exercise used to estimate VO_2_max and the second performed just after the exercise the mean percent difference for foot ESC, hand ESC and risk score were 3, 16 and 3% respectively. After one-year implementation of a health promotion program proposed to subjects with the lowest fitness level at baseline favorable changes were observed in estimated VO_2_max, waist circumference and weight (Table 2). Statistically significant changes were observed in hand and foot ESC and SUDOSCAN risk score, and these were more pronounced in people with the highest mean weekly activity (Table 3).

Thus this study is in keeping with results from previous studies that evidenced that lifestyle intervention results in beneficial metabolic changes in high-risk individuals observed among volunteers in controlled trials [16]. Importantly, the findings from this study extend the knowledge to worksite intervention programs. This is very encouraging, since many middle-aged people who are active in workforce often claim that they do not have time to participate in lifestyle intervention activities. Preventive occupational health care can offer a solution to this dilemma.

Cardio-metabolic diseases have emerged as the most common cause of death in industrialized nations [3]. Studies have also shown a notable decline in average physical activity which may have resulted in lower cardio-respiratory fitness [2]. These data suggest that physical inactivity is one of the main modifiable risk factors in the aetiology of metabolic disorders. Several studies have shown that elevated energy expenditure through daily activities is likely to be important in the primary prevention of metabolic diseases [3,4]. As such, a simple-to-use tool for evaluating cardio-respiratory fitness level to monitor and adjust an individual’s health promotion program is needed. VO_2_max assessment by an exercise test provides an accurate evaluation of cardio-respiratory fitness level but is difficult to measure due to the time required for it and poor acceptability [5]. This is especially the case for people with low fitness level who would require more intensive follow-up program and those with contra-indications such as hip and knee problems.

**Table 1:** Baseline Values for the Main Study Parameters according to SUDOSCAN Risk Score based on Sudomotor Dysfunction in Study Population by Gender.

	All		SUDOSCAN Risk Score						*p*
			No Risk		Moderate Risk		Elevated Risk		
	Mean	SD	Mean	SD	Mean	SD	Mean	SD	
**Women**	***n*** **=** **537**		***n*** **=** **162**		***n*** **=** **188**		***n*** **=** **187**		
Age (years)	49.7	8.3	42.8	7.8	51.3	6.3	54.0	6.7	<0.0001
BMI (kg/m^2^)	25.9	4.5	23.2	2.8	25.0	3.2	29.1	4.9	<0.0001
Weight (kg)	69.7	12.9	63.5	8.7	66.9	9.5	77.9	14.7	<0.0001
Waist (cm)	88.6	12.2	81.3	8.5	86.6	9.1	96.7	12.9	<0.0001
Body fat (%)	33.9	6.9	29.5ᵃ	5.7	33.0	5.7	38.7ᵇ	5.9	<0.0001*
SBP (mm Hg)	138	19	132	17	139	19	142	20	<0.0001
DBP (mm Hg)	89	11	86	10	89	11	92	10	<0.0001
HbA_1C_ (%)^&^	5.7	0.4	5.5ᵃ	0.4	5.7	0.3	6.9	0.4	<0.0001*
Estimated VO_2_max (METs)^&&^	9.1	1.8	10.3	1.7	9.0	1.5	7.9ᵇ	1.2	<0.0001*
Hand ESC (µS)	70.0	10.0	74.0	9.0	71.0	10.0	66.0	11.0	<0.0001*
Foot ESC (µS)	81.0	8.0	86.0	4.0	82.0	6.0	76.0	9.0	<0.0001*
**Men**	***n*** **=** **113**		***n*** **=** **31**		***n*** **=** **48**		***n*** **=** **34**		
Age (years)	51.2	8.2	44.4	9.4	51.9	5.8	56.5	5.1	<0.0001
BMI (kg/m^2^)	25.9	3.5	23.3	2.5	25.9	2.7	28.3	3.7	<0.0001
Weight (kg)	81.8	12.8	74.9	11.9	81.2	10.1	88.9	13.4	<0.0001
Waist (cm)	95.3	11.0	86.8	8.2	95.5	8.7	102.9	10.6	<0.0001
Body fat (%)	21.3	5.9	16.7	4.7	21.4	4.8	25.2	5.6	<0.0001
SBP	149	19	148	15	146	16	155	25	NS
DBP	92	11	91	9	93	11	93	14	NS
HbA_1C_ (%)	6.2	1.0	5.7	0.2	6.4	1.5	6.3	0.8	-
Estimated VO_2_max (METs)^&&^	12.2	2.5	13.9	2.4	12.1	2.3	10.4	1.9	<0.0001
Hand ESC (µS)	69.0	11.0	75.0	6.0	69.0	11.0	65.0ᵇ	12.0	0.0003*
Foot ESC (µS)	83.0	5.0	86.0	4.0	84.0	4.0	80.0	6.0	<0.0001*

^&^ performed on 168 women and 11 men; ^&&^ performed on 453 women and 88 men; *p*-values denote global comparisons. ESC denotes SUDOSCAN risk score value; * remained significant after adjustment on age and BMI; ᵃ not significant between no risk and moderate risk. ᵇ not significant between moderate and elevated risk; All other comparisons between groups were significant after adjustment on age and BMI; Thresholds value for the SUDOSCAN risk score: no risk ≤ 20, Moderate risk [20–30], elevated risk ≥ 30

**Table 2:** Evolution of Weight, Waist, Estimated VO_2_max and SUDOSCAN Risk Score Value in 154 Women included in the 12-month Lifestyle Intervention Programme.

	Baseline		12 months		
	Mean	SD	Mean	SD	*p*
Weight (kg)	76.4	12.5	74.9	12.3	<0.0001
Waist (cm)	95.7	11.5	93.3	11.4	<0.0001
Estimated VO_2_max (METs)^&^	7.8	1.2	8.5	1.4	<0.0001
Hand ESC (µS)	67.7	13.4	72.3	12.9	<0.0001
Foot ESC (µS)	78.1	13.2	84.1	8.3	<0.0001
SUDOSCAN risk score (%)	30.0	11.0	24.0	13.0	<0.0001

^&^ performed on 103 women

**Table 3:** Change between Baseline and 12-month Follow-up in Weight, Waist VO_2_max and SUDOSCAN Risk Score Value in 154 Women included in the 12-month Lifestyle Intervention Program according to Weekly Physical Activity Level assessed during 18 ± 8 Weeks.

	Without Follow-up of Training Level		Low Weekly Activity^&^		High Weekly Activity^&&^		
	(*n* = 72)		(*n* = 62)		(*n* = 20)		
	Mean	SD	Mean	SD	Mean	SD	*p*
Change in weight (kg)	−0.9	3.4	−1.6	4.0	−3.3	4.8	*NS*
Change in waist (cm)	−2.1	4.7	−2.4	4.5	−3.6	5.9	*NS*
Change in estimated VO_2_max (METs)	+0.5	0.9	+0.8	0.9	+1.1	1.2	*NS*
Change in hand ESC (µS)	+5.0	8.4	+3.0	9.4	+8.4	12.3	0.043
Change in foot ESC (µS)	+5.6	8.9	+4.9	8.9	+10.8	12.8	0.024
Change in SUDOSCAN risk score (%)	−5.1	5.3	−4.7	6.4	−8.5	6.8	0.027

^&^ Less than 150 min of moderate activity and 75 min of high activity; ^&&^ More than 150 min of moderate activity or 75 min of high activity; Moderate activity 3–7 METs, high activity > 7 METs

There is evidence to suggest that sudomotor dysfunction due to small-fiber injury may develop early in metabolic diseases such as diabetes and can be detected even in people with metabolic syndrome whose glucose level may only be slightly elevated [17]. The long efferent course of unmyelinated autonomic sudomotor fibers can be interrupted by central or peripheral autonomic disorders. On this basis a change in the sweat response may be a highly sensitive test in detecting small fiber neuropathy [18]. Low *et al.* using QSART that measures sweat response after stimulation by acetylcholine on the forearm, proximal leg and distal leg showed that a length-dependent neuropathy as the one observed in diabetes is typically associated with a loss in sweat volume that is maximal distally [7]. The dynamic sweat test was recently developed to measure sweat gland density, distribution of active sweat glands and sweat rate on the forearm and distal leg and was able to detect subtle functional changes occurring in the early stages of diabetic neuropathy [9]. In the same way intraepidermal nerve fiber density (IENFD) through skin biopsies is a marker of early small-fiber neuropathy in subjects with impaired glucose tolerance (IGT) [7]. All these tests are time consuming or invasive. SUDOSCAN allows a quantitative evaluation of sweat gland function based on electrochemical reaction between chloride of the sweat and stainless-steel electrodes with the application of a low DC [19]. A proof of concept study performed to compare ESC measurements in patients with cystic fibrosis and in controls has shown a correlation between conductance measurements and sweat chloride concentrations as measured by the standard sweat test method [20]. Several studies have been performed *in vitro* to evidence the physical basis of the electrochemical reaction between chlorides and stainless-steel electrodes [21,22]. Previous studies have shown that SUDOSCAN can be used to detect people with metabolic disturbances [10,11]. The reproducibility of SUDOSCAN before and just after exercise especially for foot ESC and risk score confirms the robustness of the test and mean differences observed are in accordance with previous evaluations of the reproducibility between two measurements few hours apart [10].

The correlation observed at baseline between estimated VO_2_max and SUDOSCAN risk score in this study suggests that this method could be used as an alternative to prediction of VO_2_max for fitness level evaluation. In this way there is a significant difference in estimated VO_2_max according to SUDOSCAN risk score classification in women and men that is still present in women after adjustment on age and BMI but not in men. This last result could be explained by the high variability in VO_2_max assessment and the size of the population. The involvement observed in hand and foot ESC and in SUDOSCAN risk score after lifestyle intervention is in accordance with improvement in QSART and IENFD due to small fiber regeneration as observed by Smith *et al.* in subjects with IGT after one year follow-up with diet and exercise (more that 150 min per week inducing reduction of BMI of the same range) [8]. Studies are ongoing to compare SUDOSCAN to QSART and skin biopsies. This improvement in sudomotor function observed in parallel with the improvement in estimated VO_2_max suggests that quantitative measurement of sudomotor function could also be used to monitor fitness level over time. These results observed in a middle-aged female population have to be confirmed in a larger population, including men and older people.

From this study it appears that sudomotor dysfunction as assessed by SUDOSCAN is correlated with cardio-respiratory fitness levels. This method which is rapid (< 3 min), non-invasive, quantitative and does not require preparation of the individual is well accepted by the subject and could be a useful tool for the screening of individual cardiometabolic risk and for the follow-up of lifestyle interventions in large working populations.

## 4. Conclusions

This study performed in a working population in Finland evidenced that SUDOSCAN a new simple and non-invasive method to measure sweat function can be used in the working context. There was a good correlation between SUDOSCAN results and estimated VO_2_max: −0.57 (*p* < 0.0001, 537 women). This quick, simple, non invasive and quantitative measurement reflecting small C-fiber status could be used to evaluate fitness level on a large scale and to follow subjects involved in a rehabilitation programme. Easy to understand quantitative results should improve motivation of the subjects involved in such programmes.
